# Analysis of occupational stress and its correlation with oxidative-antioxidant levels among employees of a power grid enterprise in Guangdong

**DOI:** 10.1186/s12888-022-04226-1

**Published:** 2022-09-06

**Authors:** Lingyu Zhang, Bin Liu, Linqian Zhou, Yashi Cai, Weizhen Guo, Weixu Huang, Xuehua Yan, Huifeng Chen

**Affiliations:** 1 Guangdong Province Hospital for Occupational Disease Prevention and Treatment, Guangdong Provincial Key Laboratory of Occupational Disease Prevention and Treatment, Guangzhou, 510300 Guangdong China; 2 School of Public Health, Guangzhou Medical University, Guangzhou, 511436 Guangdong China; 3 Shenzhen Luohu People’s Hospital, Shenzhen, 518000 Guangdong China; 4 School of Public Health, Southern Medical University, Guangzhou, 510515 Guangdong China

**Keywords:** Power grid enterprises, Occupational stress, Job demand-control, Effort-reward imbalance, Oxidative-antioxidant stress

## Abstract

**Background:**

Occupational stress and its health effects on occupational populations have attracted extensive attention from researchers in public health. The stressors faced by employees of power grid enterprises are increasing progressively, which is easy to cause occupational stress. The balance of the body’s oxidative-antioxidant levels plays an essential role in maintaining the body’s health status. This study aims to explore occupational stress and its correlation with oxidative-antioxidant levels in employees of a power grid enterprise.

**Methods:**

A cluster random sampling method was used to investigate the basic information of 528 employees in a power grid enterprise and investigate the two occupational stress models of employees by using the Job Content Questionnaire based on the job demand-control-support (JDC) model, and the Effort-Reward Imbalance Questionnaire based on the effort-reward imbalance (ERI) model, respectively. Peripheral blood samples were collected from the employees to measure the levels of malondialdehyde (MDA), total antioxidant capacity (TAC), and superoxide dismutase (SOD). The correlation between different models of occupational stress level and the body’s oxidation-antioxidation level was further explored.

**Results:**

The detection rate of high JDC model occupational stress was 50.6% and the detection rate of high ERI model occupational stress was 50.9%. The JDC model occupational stress was significantly associated with high-temperature and high-altitude operation, visual display terminal operation, monthly income, and exercise (all *P* < 0.05). The ERI model occupational stress was significantly associated with visual display terminal operation (all *P* < 0.05). The results of the generalized additive model showed that SOD levels had a non-linear relationship with the D/C ratio as well as the E/R ratio. With the D/C ratio close to 1, SOD levels raised rapidly. When the E/R ratio exceeded 1, the SOD level raised rapidly (all P<0.05) . TAC levels were negatively associated with the E/R ratio (*P* < 0.05).

**Conclusion:**

The detection rates of occupational stress in both models among employees in a power grid enterprise are higher. ERI model occupational stress was associated with body TAC and SOD levels, and JDC model occupational stress was associated with body SOD levels.

**Supplementary Information:**

The online version contains supplementary material available at 10.1186/s12888-022-04226-1.

## Background

In recent years, occupational stress, characterized by increased job strain and effort-reward imbalance, has been detected more frequently due to changes in working conditions and organizational forms caused by globalization, technological innovation, and digitalization [1]. As a significant occupational health problem related to personal physical and mental health as well as work efficiency, occupational stress, also regarded as job strain or work stress, is the status of long-term exposure to occupation-related stressors that affect overall health [2, 3]. Long-term occupational stress not only causes mental and psychological problems, such as anxiety, depression, and burnout [4, 5], but also increases the risk of coronary heart disease, skeletal muscle disease, and other physical diseases [6, 7], which leads to a decrease in job satisfaction [8], turnover tendency [9] and sickness absence rate of the occupational population [10]. Focusing on stressors and taking measures to reduce occupational stress in occupational populations can improve the physical and mental health of employees [2] but also lead to higher economic benefits in terms of increased productivity through reduced absenteeism [11].

There was moderate level evidence from previous studies that high job demands, low job control, high effort-reward imbalance, low relational justice, low procedural justice, role stress, bullying, low social support in the workplace, and other occupational stressors are associated with mental health [12]. Intense job demands can contribute to constant stress and ultimately emotional exhaustion [13]. The altered secretion of cortisol caused by occupational stress mobilizes the energy needed to facilitate adaptation to environmental demands and generates an adaptive load that leads to “wear and tear” of the brain and body [14]. Occupational stressor has the potential to deplete physical and mental resources, which can deplete energy and lead to mental health symptoms if not given sufficient opportunity to replenish resources [15].

The job demand-control (JDC) model and the effort-reward imbalance (ERI) model are two assessment models that have an excellent explanatory effect on occupational stress. The combination of the JDC and ERI models can explain more differences in health outcomes and provide a basis for the formulation of occupational stress interventions [16]. The JDC model assumes that a mismatch between low job control (skill utilization, decision authority, personal control, and decision-making ability) and high job demand (workload, work rhythm, conflict requests, and insufficient time to perform tasks) regulation leads to occupational stress [17]. Focusing on the role of different task characteristics and job control, the JDC model is suitable for examining psychosocial job characteristics in the workplace [15]. At present, the JDC model is mostly used for medical staff, workers, civil servants, bus drivers, and other job groups, because naturally, these jobs pose some demand and control over work. The ERI model, incorporating both situational (extrinsic) and personal (intrinsic) characteristics, assumes that effort at work should properly obtain the reward, and the difference between the two will lead to disturbing and distressing events [13]. Effort refers to the perceived needs of the worker in performing the task, such as job tasks, duties, and so on. Reward such as money, respect, job security, job recognition, career prospects, job security, status, and so on, in turn, implies the benefits that result from effort [17]. In contrast to the JDC model, the ERI model focuses on the violation of reciprocal communication between employees and employers/managers [18]. Previous studies using the ERI model mostly focus on the medical staff, polices, teachers, and other committed personnel dealing with interpersonal communication.

With the rapid development of the social economy, grid enterprises’ workload of power generation, transmission, and distribution has gradually increased [19]. Power grid enterprise employees, especially those in different positions such as management, maintenance, dispatching, and substation operation, need to adjust work tasks and deal with emergency problems according to the structure of consumer load demand [20]. Not only are they exposed to occupational hazards such as noise, high temperature and altitude, video display terminal (VDT) operation, and electromagnetic radiation (EMR), but they are also exposed to stressors such as shift work, which can lead to increased risk of occupational stress [21, 22].

Oxidative stress (OS) is the imbalance of oxidative and antioxidant levels caused by the excessive synthesis of reactive oxygen species (ROS) or the disturbance of the body’s antioxidant system, which eventually leads to adverse reactions such as apoptosis and inflammation [23]. Oxidative stress is associated with various chronic diseases and pathologies, including diabetes, neurodegenerative diseases, obesity, liver and kidney damage, DNA damage, mitochondrial dysfunction, etc. [24] Commonly used markers of oxidative stress include malondialdehyde (MDA), total antioxidant capacity (TAC), and superoxide dismutase (SOD). MDA is a biomarker of oxidative damage and is produced in vivo through the peroxidation of polyunsaturated fatty acids, which can interact with proteins and participate in the pathological development of diabetes, chronic kidney disease, and cognitive impairment [25]. The antioxidant system in the body consists of endogenous antioxidant enzymes and non-enzymatic antioxidants. As an essential endogenous antioxidant enzyme, SOD controls the levels of reactive oxygen and nitrogen species by catalyzing the dismutation of superoxide into hydrogen peroxide and oxygen [26]. TAC can be used to reflect the total ability of the body to scavenge reactive oxygen/nitric oxide enzymes by intercepting and terminating free radical chain reactions and deactivating free radicals and oxidants through non-enzymatic antioxidants in the body, including vitamin E, vitamin C, and vitamin A [27], which is a measure of the total antioxidant capacity of the body.

Studies have shown that various stressors can increase glucocorticoids by activating the hypothalamic-pituitary-adrenal (HPA) axis and that the inflammatory responses caused by stress can be dynamically regulated with oxidative stress processes to help the body adapt to different environments and maintain homeostasis in the body, which may have protective or detrimental effects [28]. There is no unified conclusion about the correlation between occupational stress and oxidative stress indicators. Studies of the ERI model occupational stress found a significant positive correlation between over-commitment and E/R ratio and MDA levels [29, 30]. Another survey of occupational stress in nurses found that the occupational stress level was negatively correlated with TAC and SOD [31]. Some studies have found a positive correlation between occupational stress and SOD [32–35]. However, some researchers did not find a significant association between TAC and occupational stress [30]. There are few studies on the correlation between occupational stress and oxidative-antioxidant levels in occupational populations, especially among employees in power grid enterprises. Therefore, the correlation between occupational stress and oxidative-antioxidant levels deserves further study. In this study, two occupational stress models were used to investigate the factors associated with the occupational stress of employees in a power grid enterprise and to analyze the correlation between occupational stress and oxidative-antioxidant levels. The results could provide a scientific basis for effective occupational stress interventions to improve employees’ physical and mental health and promote the healthy development of power grid enterprises.

## Methods

### Participants

A cluster random sampling method was used to select 528 workers from a power grid enterprise in Guangdong as the study population. Inclusion criteria for the study participants included age ≥ 18 years, working years≥1 year, no diagnosed psychiatric disorders and no family history of psychiatric disorders. The questionnaires were investigated face-to-face by trained investigators. Informed consent was obtained from participants. The study was reviewed and approved by the Committee for Medical Ethics of Guangdong Province Hospital for Occupational Disease Prevention and Treatment.

### The demographic questionnaire

The survey includes personnel number, general demographic characteristics (gender, age, marital status, education level), occupational characteristics (working years, work category, shift system, monthly income), occupational hazards (noise, electromagnetic radiation, high-temperature and high-altitude operation, visual display terminal operation) and lifestyle (smoking situation, drinking situation, exercise situation, sleeping time). Smoking was defined as cumulative smoking > 100 cigarettes [36], drinking was defined as the frequency of drinking weekly≥3 times [37], exercise was defined as days of exercise per week ≥3 days and time per exercise > 10 min [38], sleeping time was divided into short (< 6 hours), medium (6 ~ hours) and long(7 ~ hours) groups [39].

### Occupational stress survey

The Chinese version of the Job Content Questionnaire (JCQ) and the Effort-Reward Imbalance Questionnaire (ERIQ) were used to assess the JDC model and the ERI model occupational stress, respectively. Two kinds of questionnaires both used the 4-point Likert scale. Grading was scored on a scale from 1 to 4, which corresponded from ‘disagree entirely’ to ‘agree entirely’ in sequence. The JCQ contains three dimensions of job demand (5 items), job control (9 items), and social support (8 items), with a total of 22 items, The Cronbach’s alpha coefficients of three dimensions and the overall questionnaire were 0.767, 0.724, 0.912, and 0.898, respectively. The ERI contains three dimensions of effort (6 items), reward (11 items), and over-commitment (6 items), with a total of 23 items. The Cronbach’s alpha coefficients of three dimensions and the overall questionnaire were 0.841, 0.710, 0.728, and 0.863, respectively. The D/C ratio was calculated using the formula “job demand/(job control × 5/9)” [40]. The D/C ratio > 1 was defined as high JDC model occupational stress, and the D/C ratio ≤ 1 was defined as low JDC model occupational stress. The E/R ratio was calculated using “effort/(reward × 6/11)” [41]. The E/R ratio > 1 was defined as high ERI model occupational stress, and the E/R ratio ≤ 1 was defined as low ERI model occupational stress.

### Testing of oxidative stress marker levels

Two weeks before the survey, the subjects were advised to avoid excessive alcohol consumption, a high-fat diet, and medication. All subjects have fasted for 12 hours before blood sample collection. Five milliliters peripheral venous blood was drawn from all subjects when the investigation was performed. Then plasma was collected by centrifugation at 4 °C, 2000 r/min for 5 min. The levels of MDA, TAC, and SOD in plasma were measured according to the instructions for the Lipid Oxidation (MDA) Assay Kit, Total SOD Activity Assay Kit (WST-8 method), and Total Antioxidant Capacity Assay Kit (ABTS method), respectively (Beyotime Biotechnology Co., Ltd.). The levels of MDA, TAC and SOD were measured at 535 nm, 734 nm, and 450 nm respectively using an enzyme marker. The levels of each oxidative-antioxidant index were calculated based on the standard curves. MDA, TAC, and SOD levels were measured in μM, mM, and units, respectively.

### Quality control

Before the survey, the investigators are trained uniformly and introduced to the survey content and precautions for subjects. After a detailed explanation of the purpose and content of the survey by the surveyor to the respondents before the survey, face-to-face questioning is used to collect information from the study participants. The questionnaires were checked and collected on-site. Questionnaires with more than 10% of entries missing were rejected as invalid. Epidata double entry and consistency checks were carried out to ensure that the data were authentic and reliable.

### Statistical analysis

EpiData 3.1 was used to build the database, and SPSS 25.0 software as well as R version 4.0.5 was used for statistical analysis. Firstly, we examined the association between individual characteristics and the detection rate of occupational stress. By comparing rates of count data (Pearson χ^2^ test), we identified any significant variables on occupational stress. To further made summary models, binary logistic regression analysis was used to examine the association between individual characteristics and occupational stress (enter method, with an entry criterion of 0.05 and an exclusion criterion of 0.10). The logistic regression models took into account all individual characteristic variables with *P* < 0.05 in the Pearson χ^2^ test and took the occupational stress group as the dichotomous outcome variable. Dummy variables were created for multi-categorical covariates (monthly income and sleeping time). Odds ratios (ORs) and 95% confidence intervals (95% CIs) for each variable were reported.

Measurement data of oxidative-antioxidant levels were described as mean ± standard deviation. The comparison of means between two groups was performed using a t-test for two independent samples of data. The comparison of means among multiple groups was performed using ANOVA, and a two-by-two comparison between groups was performed using the Bonferroni test. To further examine the relationship between occupational stress and oxidative-antioxidant levels, we used generalized additive models (GAM) to conduct smooth curve fitting. The R package adopted in generalized additive models is the mgcv package. MDA, TAC, and SOD levels were analyzed as dependent variables, and E/R ratio and D/C ratio as independent variables respectively. The generalized cross-validation (GCV) method was used to select the number of nodes, and the cubic spline function was used to fit the curve. For the oxidative indexes that did not show a non-linear relationship with occupational stress, multiple linear regression was used to further explore variables significantly related to oxidative-antioxidant levels (stepwise method, with an entry criterion of 0.05 and an exclusion criterion of 0.10). Dependent variables were oxidative-antioxidant levels and independent variables with *P* < 0.05 in t-test or ANOVA. The results were reported by Beta and 95% confidence intervals (95% CIs) for each variable. The test level α = 0.05 (two-sided).

## Results

### Demographic characteristics

Among the 528 study subjects, 463 (87.7%) were male, and 65 (12.3%) were female. The average age was (39.04 ± 9.94) years old, and the average working years was (17.26 ± 11.21) years. More people were married, accounting for 76.1%. And 52.3% of those with an educational level of undergraduate. The work categories were mainly in substation operation and maintenance, accounting for 44.5 and 35.4%, respectively. There were 373 shift workers, accounting for 70.6%. Employees’ monthly income was mainly 5000 ~ and 7000 ~ yuan, accounting for 39 and 33%, respectively. Electromagnetic radiation exposure and high-temperature and high-altitude operation were more numerous, accounting for 62.5 and 62.7%, respectively. In comparison, noise exposure and visual display terminal operation accounted for 33.9 and 33.3%, respectively. The proportion of employees who smoke, drink and exercise is higher, accounting for 30.3, 65.5, and 75.6%, respectively. Most of the employees’ sleeping time was 6 ~ hours, accounting for 46.4%.

### Occupational stress

As shown in Table 1, the detection rate of high occupational stress was 50.6% (267/528) for the JDC model and 50.9% (269/528) for the ERI model among the 528 study participants. In the JDC model, the differences in the detection rates of occupational stress between the groups of monthly income, high-temperature and high-altitude operation, visual display terminal operation, exercise situation, and sleeping time were all statistically significant (all *P* < 0.05). In the ERI model, the differences in the detection rates of occupational stress between the groups of gender, age, marital status, working years, noise, electromagnetic radiation, high-temperature and high-altitude operation and, visual display terminal operation were all statistically significant (all *P* < 0.05).

**Table 1:** Comparison with detection rates of occupational stress among employees with different individual characteristics

Group	Total (*n* = 528)	JDC model occupational stress				ERI model occupational stress			
	N (%)	D/C > 1 (*n* = 267,50.6%)	Percentage (%)	*x*^*2*^ value	*P* value	E/R > 1 (*n* = 269,50.9%)	Percentage (%)	*x*^*2*^ value	*P* value
Gender				1.198	0.274			3.893	0.048
Male	463(87.7)	230	49.7			243	52.5		
Female	65(12.3)	37	56.9			26	40.0		
Age/years				4.055	0.256			31.189	< 0.001
20~	109(20.6)	50	45.9			31	28.4		
30~	158(29.9)	85	53.8			96	60.8		
40~	168(31.8)	91	54.2			96	57.1		
50~	93(17.7)	41	44.1			46	49.5		
Marital Status				2.243	0.134			9.628	0.002
Single	126(23.9)	61	48.4			49	38.9		
Married	402(76.1)	206	51.2			220	54.7		
Education level				1.648	0.649			4.858	0.183
High School and below	56(10.6)	24	42.9			27	48.2		
Junior college	129(24.4)	66	51.2			73	56.6		
Undergraduate	276(52.3)	141	51.1			130	47.1		
Master and above	67(12.7)	36	53.7			39	58.2		
Working years/years				2.191	0.534			19.678	< 0.001
< 10	188(35.6)	89	47.3			75	39.9		
10~	105(19.9)	61	58.1			64	61.0		
20~	144(27.3)	74	51.4			87	60.4		
30~	91(17.2)	43	47.3			43	47.3		
Work category				4.947	0.293			7.848	0.097
Management	10(1.9)	6	60.0			5	50.0		
Maintenance	187(35.4)	91	48.7			99	52.9		
Dispatching	30(5.7)	12	40.0			19	63.3		
Substation operation	235(44.5)	129	54.9			122	51.9		
Other	66(12.5)	29	43.9			24	36.4		
Shift system				0.120	0.729			1.807	0.179
Day shift	373(70.6)	189	50.7			183	49.1		
Shift work	155(29.4)	78	50.3			86	55.5		
Monthly income/yuan				9.478	0.024			7.430	0.059
< 5000	96(18.2)	38	39.6			42	43.8		
5000~	206(39.0)	100	48.5			98	47.6		
7000~	174(33.0)	95	54.6			96	55.2		
9000~	52(9.8)	34	65.4			33	63.5		
Noise exposure				2.433	0.119			9.551	0.002
No	349(66.1)	168	48.1			161	46.1		
Yes	179(33.9)	99	55.3			108	60.3		
Electromagnetic radiation exposure				1.213	0.271			10.331	0.001
No	198(37.5)	94	47.5			83	41.9		
Yes	330(62.5)	173	52.4			186	56.4		
High-temperature and high-altitude operation				18.071	< 0.001			6.686	0.010
No	197(37.3)	76	38.6			86	43.7		
Yes	331(62.7)	191	57.7			183	55.3		
Visual display terminal operation				13.638	< 0.001			18.567	< 0.001
No	352(66.7)	158	44.9			156	44.3		
Yes	176(33.3)	109	61.9			113	64.2		
Smoking				0.343	0.558			3.228	0.072
No	368(69.7)	183	49.7			178	48.4		
Yes	160(30.3)	84	52.5			91	58.9		
Drinking				2.738	0.098			3.172	0.075
No	182(34.5)	83	45.6			83	45.6		
Yes	346(65.5)	184	53.2			186	53.8		
Exercise				5.682	0.017			2.144	0.285
No	129(24.4)	77	59.7			71	55.0		
Yes	399(75.6)	190	47.6			198	49.6		
Sleeping time/h				6.337	0.042			5.243	0.073
< 6	157(29.7)	73	46.5			85	54.1		
6~	245(46.4)	132	53.9			131	53.5		
7~	126(23.9)	51	40.5			53	42.1		

To examine the association between sociodemographic variables and occupational stress in two different models, binary logistic regression analysis was performed by taking JDC and ERI occupational stress as dependent variables and the variables with the statistical difference (*P* < 0.05) in Table 1 as independent variables. The results of the analysis were shown in Tables 2 and 3. In the JDC model, high-temperature and high-altitude operation (OR = 2.168, 95% CI: 1.489 to 3.158), visual display terminal operation (OR = 1.815, 95% CI: 1.234 to 2.669), and exercise (OR = 0.591, 95% CI: 0.386 to 0.905) were significantly associated with occupational stress among the employees. Compared with the monthly income group of < 5000 yuan, both the monthly income group of 7000 ~ yuan (OR = 1.853, 95% CI: 1.093 to 3.142) and 9000 ~ yuan (OR = 3.031, 95% CI: 1.453 to 6.321) significantly increased the risk of JDC model occupational stress. In the ERI model, visual display terminal operation (OR = 2.146, 95% CI: 1.450 to 3.176) significantly increased the risk of occupational stress among the employees.

**Table 2:** Logistic regression analysis for factors associated with the JDC model occupational stress among employees

Variables	*Beta*	S.E	Wald	*P* value	*OR*(95%*CI*)
Monthly income/yuan			10.026	0.018	
<5000	reference				
5000~	0.416	0.263	2.499	0.114	1.516 (0.905,2.540)
7000~	0.617	0.269	5.239	0.022	1.853 (1.093.3.142)
9000~	1.109	0.375	8.740	0.003	3.031 (1.453.6.321)
High-temperature and high-altitude operation					
No	reference				
yes	0.774	0.192	16.277	< 0.001	2.168 (1.489,3.158)
Visual display terminal operation					
No	reference				
yes	0.596	0.197	9.185	0.002	1.815 (1.234,2.669)
Exercise					
No	reference				
yes	−0.526	0.217	5.854	0.016	0.591 (0.386,0.905)
Sleeping time/h			4.651	0.098	
< 6	reference				
6~	0.145	0.215	0.452	0.501	1.156 (0.758,1.763)
7~	−0.358	0.255	1.975	0.160	0.699 (0.424,1.152)

Dependent variable assignment: JDC model occupational stress: low = 0, high = 1; independent variable assignment: high-temperature and high-altitude operation, visual display terminal operation, exercise: no = 0, yes = 1; Monthly income (dummy variable): < 5000 yuan = 1 (reference), 5000 ~ yuan = 2, 7000 ~ yuan = 3, 9000 ~ yuan = 4; Sleep time (dummy variable): < 6 hours = 1 (reference), 6 ~ hours = 2, 7 ~ hours = 3

**Table 3:** Logistic regression analysis for factors associated with the ERI model occupational stress among employees

Variables	*Beta*	S.E	Wald	*P* value	*OR*(95%*CI*)
Gender					
Male	reference				
Female	−0.401	0.285	1.976	0.160	0.670 (0.383,1.171)
Age/years	0.035	0.031	1.251	0.263	1.036 (0.974,1.102)
Working years/years	−0.015	0.027	0.305	0.580	0.985 (0.935,1.039)
Married status					
Single	reference				
Married	0.404	0.252	2.584	0.108	1.499 (0.915,2.454)
Noise exposure					
No	reference				
yes	0.346	0.213	2.649	0.104	1.414 (0.932,2.145)
Electromagnetic radiation exposure					
No	reference				
yes	0.340	0.204	2.785	0.095	1.405 (0.942, 2.096)
High-temperature and high-altitude operation					
No	reference				
yes	0.283	0.200	2.006	0.157	1.327 (0.897,1.963)
Visual display terminal operation					
No	reference				
yes	0.764	0.200	14.575	< 0.001	2.146 (1.450,3.176)

Dependent variable assignment: ERI model occupational stress: low = 0, high = 1; independent variable assignment: gender: male = 1; female = 2; marital status: single = 1, married = 2; noise exposure, electromagnetic radiation exposure, high-temperature and high-altitude operation, visual display terminal operation, exercise: no = 0, yes = 1; Age and working years are continuous variables and not assigned a value

### Oxidative-antioxidant level

The mean level of MDA, TAC, and SOD of the 528 participants was 1.907 ± 1.231 μM, 1.288 ± 0.175 mM, and 0.354 ± 0. 095 units, respectively. As shown in Table 4, the differences in MDA levels among workers of different age, marital status, education, working years, and EMR exposure were statistically significant (all *P* < 0.05). The differences in TAC levels among workers of different age, smoking, and drinking were statistically significant (all *P* < 0.05).

**Table 4:** The results of oxidative-antioxidation levels in employees with different individual characteristics

Group	MDA (μM)			TAC(mM)			SOD (units)		
	Mean ± SD	*t/F* value	*P* value	Mean ± SD	*t/F* value	*P* value	Mean ± SD	*t/F* value	*P* value
Gender		−1.501	0.138		−0.333	0.740		−0.024	0.981
Male	1.870 ± 1.176			1.287 ± 0.177			0.354 ± 0.094		
Female	2.171 ± 1.556			1.295 ± 0.159			0.354 ± 0.106		
Age/years		6.168	< 0.001		2.905	0.034		1.681	0.170
20~	1.815 ± 1.149			1.317 ± 0.176			0.337 ± 0.090		
30~	1.734 ± 1.107			1.274 ± 0.190			0.360 ± 0.093		
40~	2.238 ± 1.393^abc^			1.301 ± 0.142			0.353 ± 0.104		
50~	1.719 ± 1.109			1.253 ± 0.193^a^			0.363 ± 0.087		
Marital Status		−2.595	0.010		1.594	0.112		−0.622	0.534
Single	1.697 ± 0.945			1.309 ± 0.184			0.349 ± 0.100		
Married	1.973 ± 1.302			1.281 ± 0.171			0.355 ± 0.094		
Education level		3.313	0.020		1.922	0.125		0.580	0.629
High School and below	1.770 ± 1.241			1.236 ± 0.201			0.345 ± 0.095		
Junior college	2.126 ± 1.359^d^			1.298 ± 0.155			0.347 ± 0.088		
Undergraduate	1.915 ± 1.220			1.293 ± 0.180			0.358 ± 0.097		
Master and above	1.570 ± 0.901			1.288 ± 0.157			0.358 ± 0.103		
Working years/years		4.285	0.005		1.012	0.387		1.764	0.153
< 10	1.759 ± 1.149			1.293 ± 0.180			0.345 ± 0.090		
10~	2.005 ± 1.255			1.298 ± 0.178			0.348 ± 0.100		
20~	2.168 ± 1.369^ef^			1.292 ± 0.158			0.368 ± 0.102		
30~	1.693 ± 1.063			1.259 ± 0.185			0.356 ± 0.088		
Work category		2.368	0.052		1.915	0.107		2.166	0.072
Management	1.877 ± 1.141			1.213 ± 0.218			0.375 ± 0.105		
Maintenance	2.088 ± 1.374			1.269 ± 0.170^i^			0.358 ± 0.090		
Dispatching	1.601 ± 0.992			1.278 ± 0.231			0.395 ± 0.115		
Substation operation	1.771 ± 1.108			1.296 ± 0.175			0.345 ± 0.097		
Other	2.025 ± 1.275			1.325 ± 0.146			0.351 ± 0.091		
Shift system		0.043	0.966		−1.417	0.157		−0.248	0.804
Day shift	1.909 ± 1.219			1.281 ± 0.176			0.353 ± 0.098		
Shift work	1.904 ± 1.265			1.304 ± 0.171			0.356 ± 0.089		
Monthly income/yuan		0.578	0.630		1.256	0.289		0.988	0.398
< 5000	1.858 ± 1.346			1.314 ± 0.164			0.346 ± 0.105		
5000~	1.988 ± 1.285			1.273 ± 0.166			0.349 ± 0.087		
7000~	1.831 ± 1.064			1.292 ± 0.180			0.361 ± 0.090		
9000~	1.937 ± 1.325			1.282 ± 0.203			0.364 ± 0.123		
Noise exposure		0.856	0.392		0.566	0.572		0.228	0.820
No	1.940 ± 1.244			1.291 ± 0.167			0.355 ± 0.093		
Yes	1.843 ± 1.206			1.282 ± 0.189			0.353 ± 0.101		
Electromagnetic radiation exposure		3.552	< 0.001		0.841	0.401		0.359	0.720
No	2.165 ± 1.393			1.296 ± 0.180			0.356 ± 0.093		
Yes	1.753 ± 1.096			1.283 ± 0.171			0.353 ± 0.097		
High-temperature and high-altitude operation		−1.471	0.142		0.872	0.384		−1.176	0.240
No	1.810 ± 1.085			1.296 ± 0.181			0.348 ± 0.093		
Yes	1.965 ± 1.309			1.283 ± 0.171			0.358 ± 0.097		
Visual display terminal operation		−0.510	0.611		1.083	0.280		−1.040	0.299
No	1.888 ± 1.188			1.294 ± 0.164			0.351 ± 0.094		
Yes	1.946 ± 1.315			1.275 ± 0.194			0.360 ± 0.099		
Smoking		1.116	0.265		2.272	0.023		−0.771	0.441
No	1.947 ± 1.279			1.299 ± 0.171			0.352 ± 0.101		
Yes	1.817 ± 1.112			1.262 ± 0.181			0.358 ± 0.081		
Drinking		−0.246	0.806		2.691	0.007		−1.410	0.159
No	1.889 ± 1.282			1.314 ± 0.159			0.346 ± 0.094		
Yes	1.917 ± 1.205			1.273 ± 0.181			0.358 ± 0.096		
Exercise		0.198	0.843		1.642	0.101		−0.178	0.859
No	1.913 ± 1.204			1.295 ± 0.171			0.354 ± 0.092		
Yes	1.889 ± 1.316			1.266 ± 0.185			0.355 ± 0.106		
Sleeping time/h		0.500	0.607		0.228	0.796		2.824	0.060
< 6	1.862 ± 1.162			1.280 ± 0.172			0.363 ± 0.103		
6~	1.965 ± 1.288			1.291 ± 0.178			0.357 ± 0.090		
7~	1.852 ± 1.206			1.292 ± 0.173			0.337 ± 0.094		

Compared with the age group 20 ~ years old

^a^*P* < 0.05; compared with the age group 30 ~ years old

^b^*P* < 0.05; compared with the age group 50 ~ years old

^c^*P* < 0.05; compared with the group of master or above

^d^*P* < 0.05; compared with the work year group < 10 years

^e^*P* < 0.05; compared with the work year group 30 ~ years

^f^*P* < 0.05

In both JDC and ERI models, the serum SOD levels of employees in the high occupational stress group were higher than those in the low occupational stress group, and the differences were statistically significant (*P* < 0.05). In the ERI model, the serum TAC levels of employees in the high occupational stress group were lower than those in the low occupational stress group, and the differences were statistically significant (*P* < 0.05). See Table 5.

**Table 5:** Comparison of oxidative-antioxidation levels of employees with different levels of occupational stress

Group	MDA(μM)		TAC(mM)		SOD (units)	
	Mean ± SD	*P* value	Mean ± SD	*P* value	Mean ± SD	*P* value
JDC model		0.143		0.551		< 0.001
Low occupational stress	1.828 ± 1.092		1.292 ± 0.181		0.328 ± 0.088	
High occupational stress	1.985 ± 1.351		1.283 ± 0.169		0.379 ± 0.095	
ERI model		0.497		0.014		< 0.001
Low occupational stress	1.944 ± 1.221		1.307 ± 0.156		0.330 ± 0.090	
High occupational stress	1.872 ± 1.242		1.270 ± 0.189		0.377 ± 0.095	

**Fig. 1:**
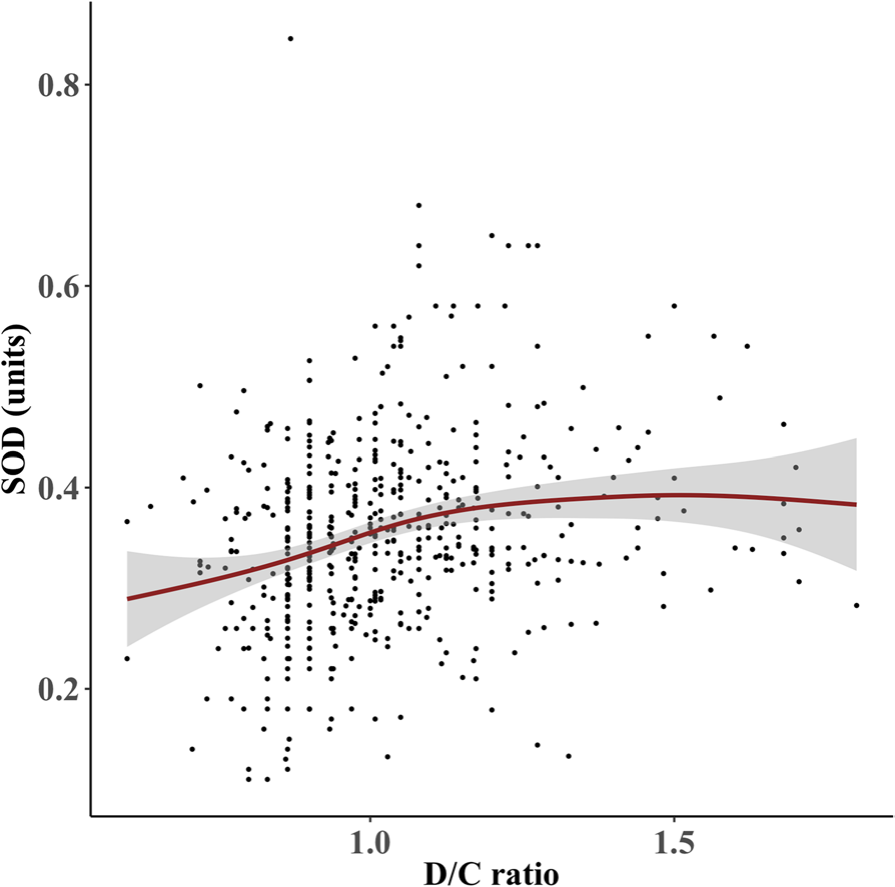
Smooth curve fitting by the generalized additive model of SOD level and D/C ratio

**Fig. 2:**
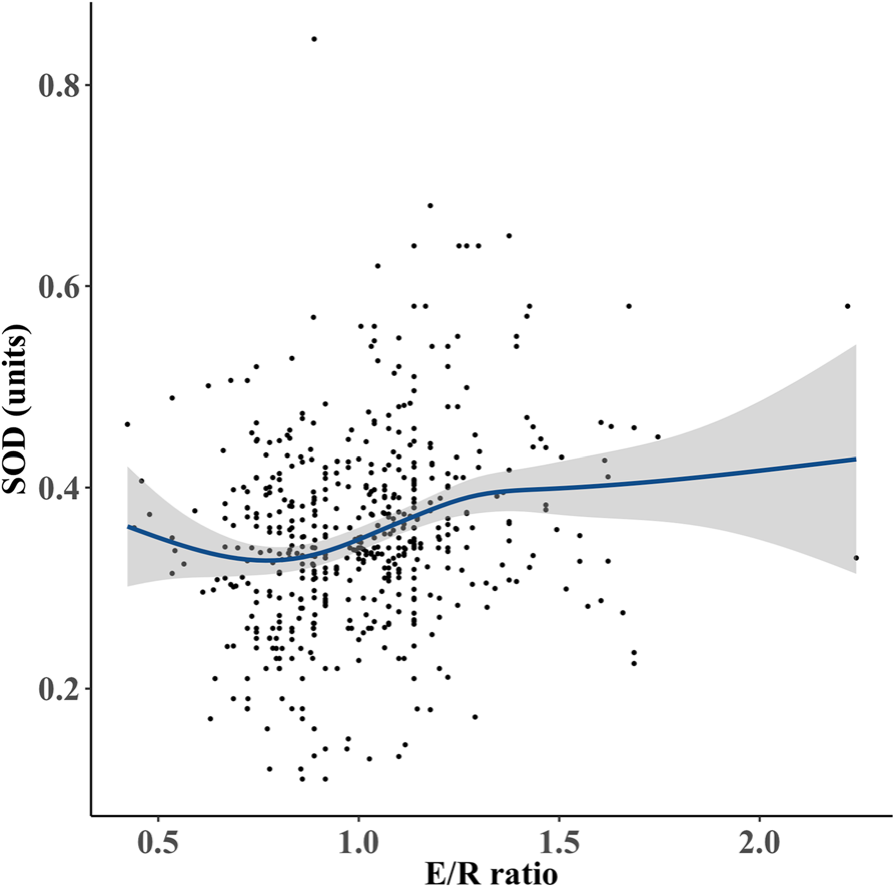
Smooth curve fitting by the generalized additive model of SOD level and E/R ratio

The results of generalized additive models showed there were non-linear relationships between SOD with E/R ratio as well as D/C ratio, and the difference was statistically significant (*P* < 0.05). The non-linear relationships between TAC with E/R ratio are approximately statistically significant (*P* = 0.04) (Fig. S1). However, there were no non-linear relationships between TAC and D/C ratio, MDA and E/R ratio, as well as MDA and D/C ratio, without statistically significant difference (*P* = 0.16, *P* = 0.12, *P* = 0.56) (Figs. S2, S3 and S4). As shown in Fig. 1, the relationship between the SOD and D/C ratio is in an inverted L shape. When the D/C ratio is close to 1.0, SOD increases rapidly, and then tends to be flat. As shown in Fig. 2, when the E/R ratio was in the range of 0.5–1.0, SOD levels showed a U-shape. When the E/R ratio exceeded 1, SOD levels rose rapidly and then slowed down and reached a plateau. Compared with linear models of SOD levels about D/C ratio and E/R ratio respectively, nonparametric regression models constructed using the generalized additive models showed better effects, with statistically significant differences (*P* < 0.05).

**Fig. 3:**
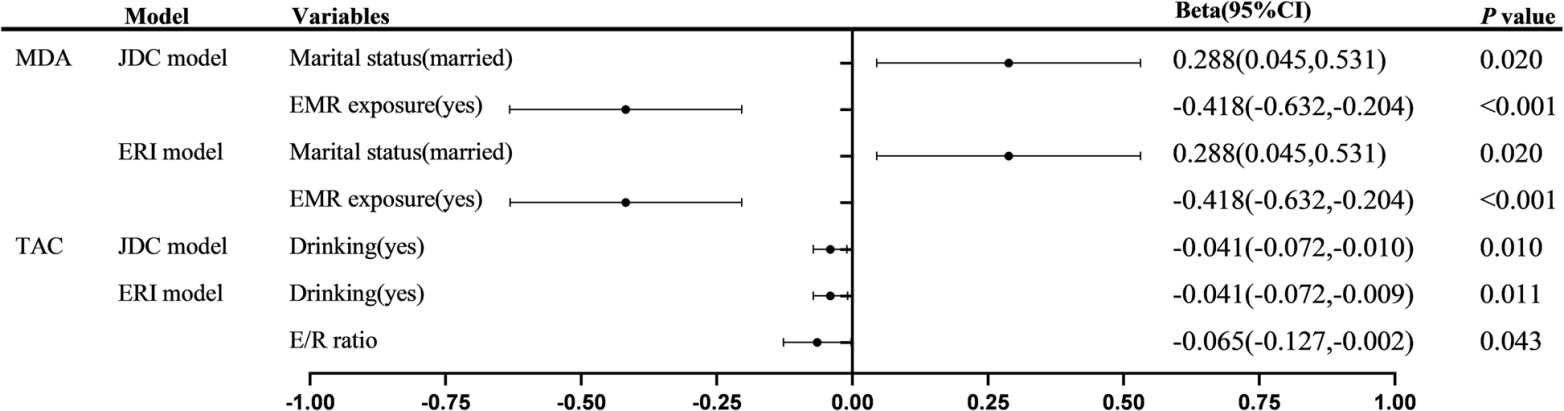
Multiple linear regression analysis of factors associated with MDA and TAC levels of employees. Note: Independent variables assigned: marriage: single = 1, married = 2; EMR exposure, drinking: no = 0, yes = 1; E/R ratio are all continuous variables and are not assigned a value

The results of multiple linear regression analysis were shown in Fig. 3. In the stepwise regression model to test the correlation between D/C ratio or E/R ratio (independent variable) and MDA (dependent variable), we additionally adjusted variables as follows (with *P* < 0.05 in Table 4): age, marital status, educational level, working years, and electromagnetic radiation exposure. In stepwise regression models using MDA levels as the dependent variable, D/C ratio and E/R ratio were neither allowed to enter the models. While married (β = 0.288, 95% CI: 0.045 to 0.531) and EMR exposure (β = − 0.418, 95% CI: − 0.632 to − 0.204) were associated with the MDA levels. In the stepwise regression model to test the correlation between D/C ratio or E/R ratio (independent variable) and TAC (dependent variable), we additionally adjusted variables as follows (with *P* < 0.05 in Table 4): age, smoking, and drinking. In the model to test the correlation between TAC levels and JDC model occupational stress, the D/C ratio was neither allowed to enter the models, while drinking (β = − 0.041, 95% CI: − 0.072 to − 0.010) was correlated with the TAC levels. In the model to test the correlation between TAC levels and ERI model occupational stress, E/R ratio (β = − 0.065, 95% CI: − 0.127 to − 0.002) and drinking (β = − 0.041, 95% CI: − 0.072 to − 0.009) was correlated with the TAC levels.

## Discussion

Among occupational populations, general demographic characteristics, occupational characteristics, occupational hazards, and lifestyle all have potential impacts on occupational health. We conduct research on occupational stress as well as oxidative-antioxidant levels in specific occupational groups, and various variables on these aspects were looked at. In this case, a significant result may be obtained by chance by multiple comparisons. To avoid the statistical bias caused by the difference of variables in the multivariate analysis, we used the conservative Bonferroni test for a two-by-two comparison of means among multiple groups, performed smooth curve fitting by the generalized additive model, and only included significant variables in the univariate analysis as covariates to adjust the multivariate model.

The study result reveals that the detection rate of JDC and ERI model occupational stress among employees in a power grid enterprise was 50.6 and 50.9%, respectively. Our results were higher than Li et al. [19], who reported in 2015 that the detection rate of JDC and ERI model occupational stress among workers in a power supply was 28.7 and 19.4%, respectively. The possible explanations could be the increasing demand for power supply, the heavier workload of power supply grid enterprises, and the increase in stressors, which may affect employees’ mental health. In the present study, high-temperature and high-altitude operation, visual display terminal operation, monthly income, and exercise situation were significantly associated with JDC model occupational stress. Statistically significant associations were also found between the visual display terminal operation and ERI model occupational stress among employees in a power grid enterprise.

Martinez et al. [22] used the Work Stress Scale (WSS) to survey workers’ stress from a utility electric power company in Brazil and found that regular exercise and monthly income were negatively associated with stress levels, which is consistent with the findings of our study that exercise reduces JDC model occupational stress. It is possibly because exercise can reduce occupational stress by various mechanisms, such as decreased neural activity and increased parasympathetic activity, suggesting that exercise may help decrease stress levels. Our study found that the risk of JDC model occupational stress was higher in the group with higher monthly income. This may be due to differences in regional, time, and job content, the higher the monthly income of employees in our study, the higher rank in the enterprise, and more responsibility they possibly performed than just technical employees, thus increasing the risk of occupational stress. Our study found that high-temperature and high-altitude operations can increase the risk of occupational stress among employees in power grid enterprises, which may be related to significantly higher job demands. In addition, employees in positions such as maintenance and dispatch often work outdoors at high temperatures and altitudes, where changes in temperature and height can affect the expression levels and dynamic balance of neurotransmitters such as serotonin and dopamine in the brain [42]. As a lesser concern occupational hazard, visual display terminal operation often requires employees to maintain the same posture for long periods, focusing on the same position on the screen and the work content is repetitive and monotonous, often causing occupational stress, musculoskeletal problems, visual fatigue, and anxiety [43].

Our study found that MDA levels were significantly associated with marital status and electromagnetic radiation exposure and TAC levels were significantly associated with drinking. However, there were no statistically significant differences between the two models of occupational stress and MDA levels among employees in a power grid enterprise. Salem et al. [30] surveyed on the nurses’ population found a significant positive correlation between MDA levels and ERI model occupational stress. Previous studies found that due to the secretion of testosterone, aging, and other factors, males and young people have significantly lower MDA levels than females and old people, respectively [44, 45]. The difference between our study and Salem’s study may be because the subjects of our study were employees of a power grid enterprise, and a relatively large proportion of the study population was 40–50. In contrast, the population studied by Salem et al. was mainly a female nurse group aged 20–40 years.

Our results showed an approximately statistically significant non-linear relationship between the ERI model occupational stress and TAC levels(*P* = 0.04). Multiple linear regression analysis found a negative association between the ERI model occupational stress and TAC levels (*P* = 0.043), but this was much less significant than the correlation between the E/R ratio and SOD. This may be because our questionnaires did not contain dietary status factors to adjust for the relationship between occupational stress and TAC levels, leading to underestimation or overestimation of the association between exposure and outcome. Although non-enzymatic antioxidants are involved in maintaining redox homeostasis, the TAC levels may be reduced due to the dietary habits and composition of employees who were unable to supplement with antioxidants in a timely manner [26].

In addition to non-enzymatic antioxidants such as endogenous substances like bilirubin and nutritional compounds such as tocopherols, antioxidant enzymes such as SOD, as important antioxidant indicators, can enhance cellular defense by effectively neutralizing increased levels of oxidation in the body [26]. And another study suggests that environmental and psychological stress may promote inflammatory responses by activating the hypothalamic-pituitary-adrenal (HPA) axis to increase cortisol levels, thereby causing changes in oxidative stress levels and increasing SOD levels [28]. In this study, the results of generalized additive models and smooth curve fitting shown with the increase of D/C ratio or E/R ratio, SOD levels rose rapidly when employees of the power grid enterprise shifted from the low occupational stress group to the high occupational stress group in particular. This suggests a greater change in the body’s oxidative stress changes when employees shift from low occupational stress to high occupational stress. Casado et al. [32–34] found higher levels of SOD activity in night shift nurses, palliative care workers, and prehospitalary emergency service, who had high job demand, stress, and burnout levels, suggesting that high occupational stress is associated with increased SOD levels, consistent with our study. Zhu et al. used the Occupational Stress Inventory (OSI) to investigate 172 nurses and found that SOD levels were positively associated with job risk and negatively associated with tolerance [35]. This suggests increasing job risk, which decreased job security and rewards as well as increased ERI occupational stress levels, was possibly associated with increasing SOD levels of employees in a power grid enterprise. For another, decreasing tolerance, which decreased job control and increased JDC occupational stress levels, was also probably associated with increasing SOD levels of subjects. Therefore, our study suggests that two models of occupational stress can cause oxidative stress among employees in a power grid enterprise and that antioxidant enzymes such as SOD, as the first line of defense, may neutralize oxidative damage in the body by increasing its expression level.

There are some limitations. For example, this study is a cross-sectional study of a power grid enterprise, and it is difficult to determine the causal association between occupational stress and oxidative stress levels. In addition, this study did not include a survey on diet, which may affect the analysis of the results. And this information needs to be considered in subsequent studies on occupational stress and oxidative-antioxidant levels in large samples.

## Conclusion

In summary, monthly income, exercise, high-temperature, and high-altitude operation, and video display terminal operation are factors associated with the occupational stress of employees in power grid enterprises. JDC model occupational stress, ERI model occupational stress, drinking, EMR, and marital status are correlated with oxidative-antioxidant levels. With the development of power grid enterprises, the impact of changes in the working environment and job content on the mental health of workers may lead to changes in TAC and SOD levels. Power grid enterprises should introduce advanced equipment, improve the level of automation, reduce the difficulty of operation, and at the same time provide and ensure the supply to employees with cooling, anti-electromagnetic radiation, visual fatigue relief, and other labor protection appliance. It is also vital for enterprises to focus on employees’ work difficulties and mental health, especially high-income earners, which need to arrange regular medical check-ups and training on mental health for employees. Employees should also maintain moderate exercise, combine work and rest, reduce alcohol consumption, eat a balanced diet, and take timely supplements of foods rich in antioxidants.

There are few studies on the correlation between occupational stress and oxidative stress levels. Based on the occupational populations of power grid enterprise employees, our study comprehensively analyzes the correlation between two models occupational stress and oxidative stress from demographic characteristics, occupational characteristics, and lifestyle, especially in occupational hazardous. It could provide a scientific basis for early intervention and health risk assessment research of occupational stress in the occupational populations in power grid industry.

## Supplementary Information

**Additional file 1: Fig. S1.** Smooth curve fitting by the generalized additive model of TAC level and E/R ratio.

**Additional file 2: Fig. S2.** Smooth curve fitting by the generalized additive model of TAC level and D/C ratio.

**Additional file 3: Fig. S3.** Smooth curve fitting by the generalized additive model of MDA level and E/R ratio.

**Additional file 4: Fig. S4.** Smooth curve fitting by the generalized additive model of MDA level and D/C ratio.

## Data Availability

The data that support the findings of this study are available on request from the corresponding author.

## Abbreviations

EMR: Electromagnetic radiation
ERI: Effort-reward imbalance
ERIQ: Effort-Reward Imbalance Questionnaire
HPA: Hypothalamic-pituitary-adrenal
JCQ: Job Content Questionnaire
JDC: Job demand-control
MDA: Malondialdehyde
OR: Odds Ratio
OS: Oxidative stress
ROS: Reactive oxygen species
SOD: Superoxide dismutase
TAC: Total antioxidant capacity
VDT: Video display terminal
