# Supplementary figures and images for: Analysis of occupational stress and its correlation with oxidative-antioxidant levels among employees of a power grid enterprise in Guangdong
Source: BMC Psychiatry. 2022 Sep 6;22:593. doi: 10.1186/s12888-022-04226-1 (PMC9446777; doi:10.1186/s12888-022-04226-1)

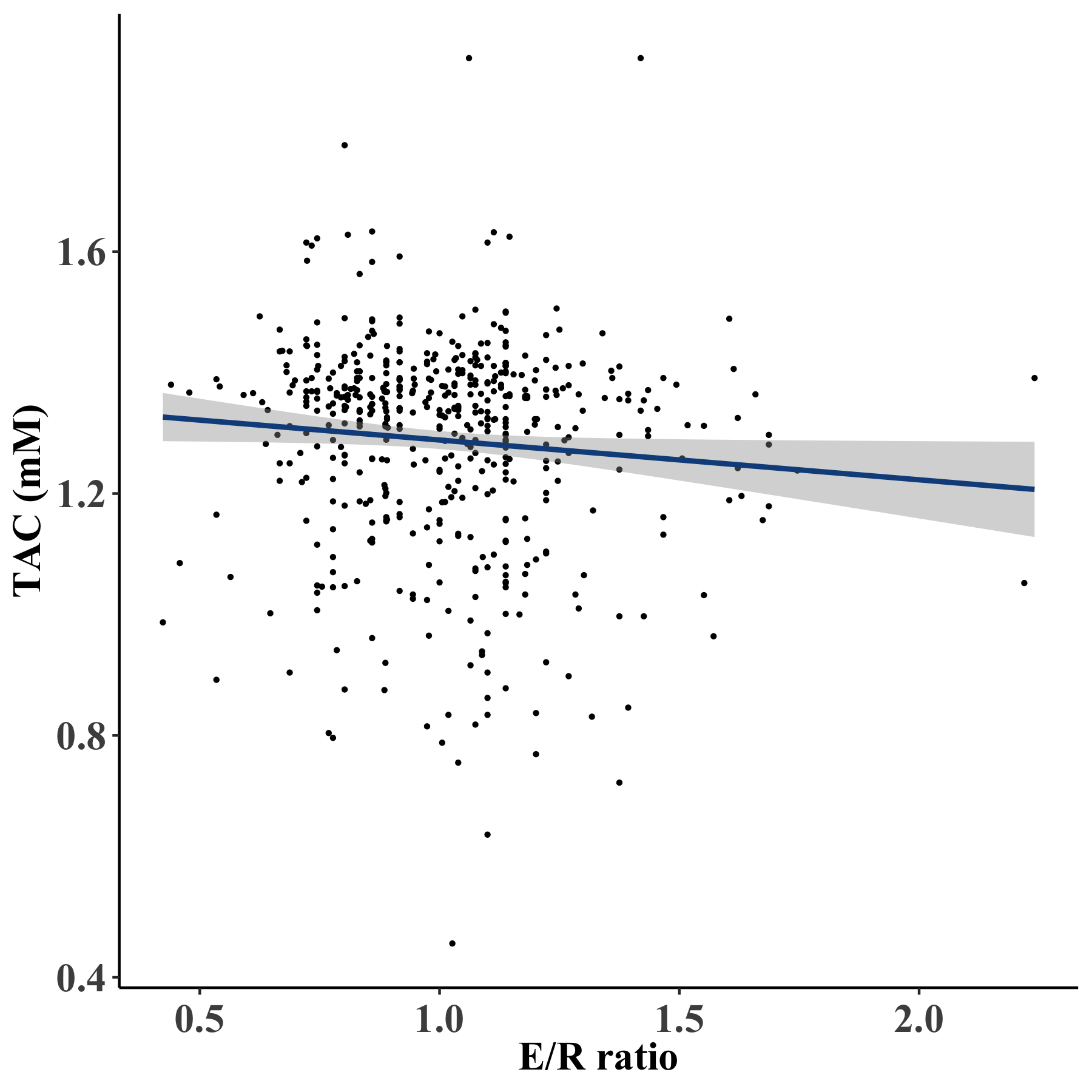

Supplement: Supplementary file 1 — Additional file 1: Fig. S1. Smooth curve fitting by the generalized additive model of TAC level and E/R ratio. [file 12888_2022_4226_MOESM1_ESM.tif]

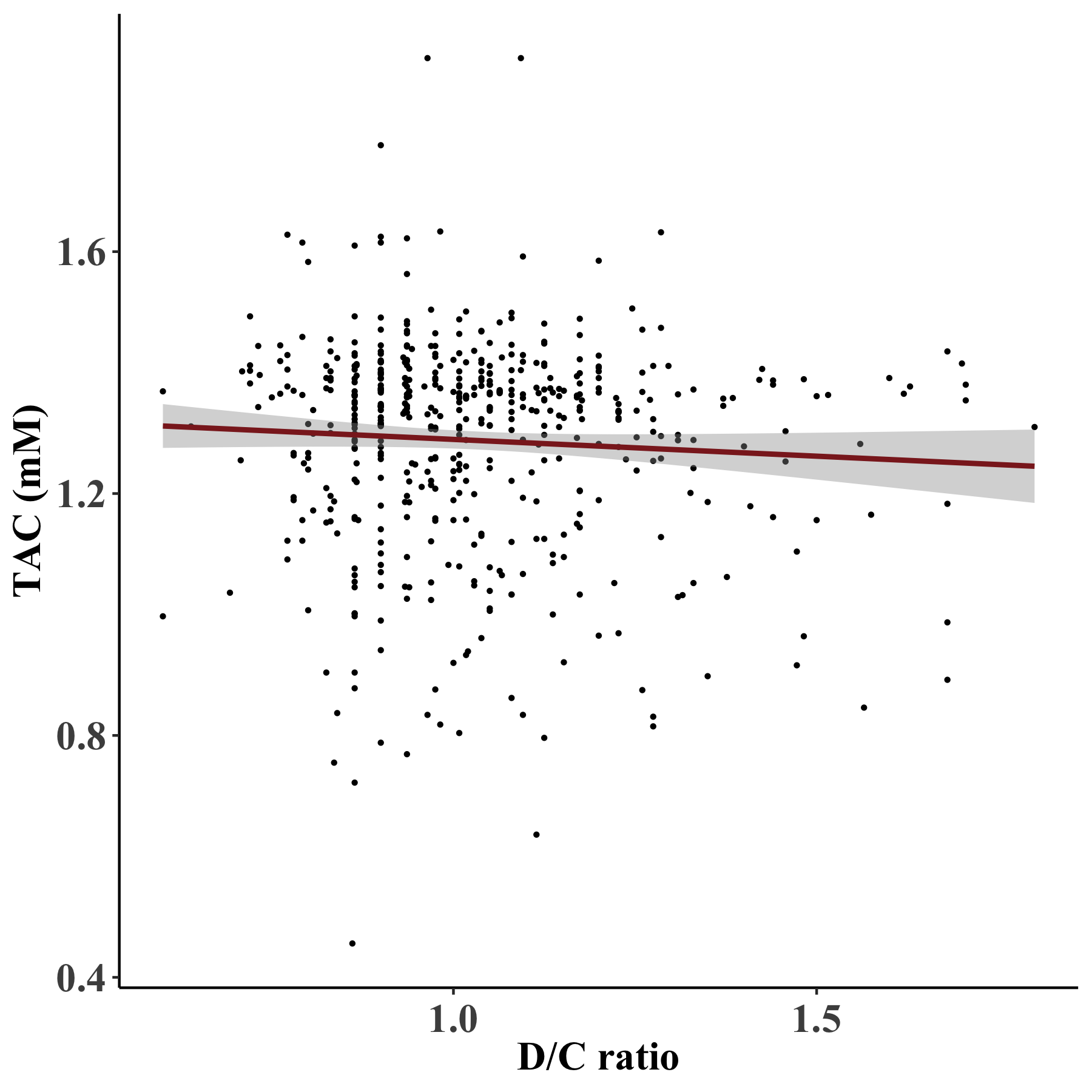

Supplement: Supplementary file 2 — Additional file 2: Fig. S2. Smooth curve fitting by the generalized additive model of TAC level and D/C ratio. [file 12888_2022_4226_MOESM2_ESM.tif]

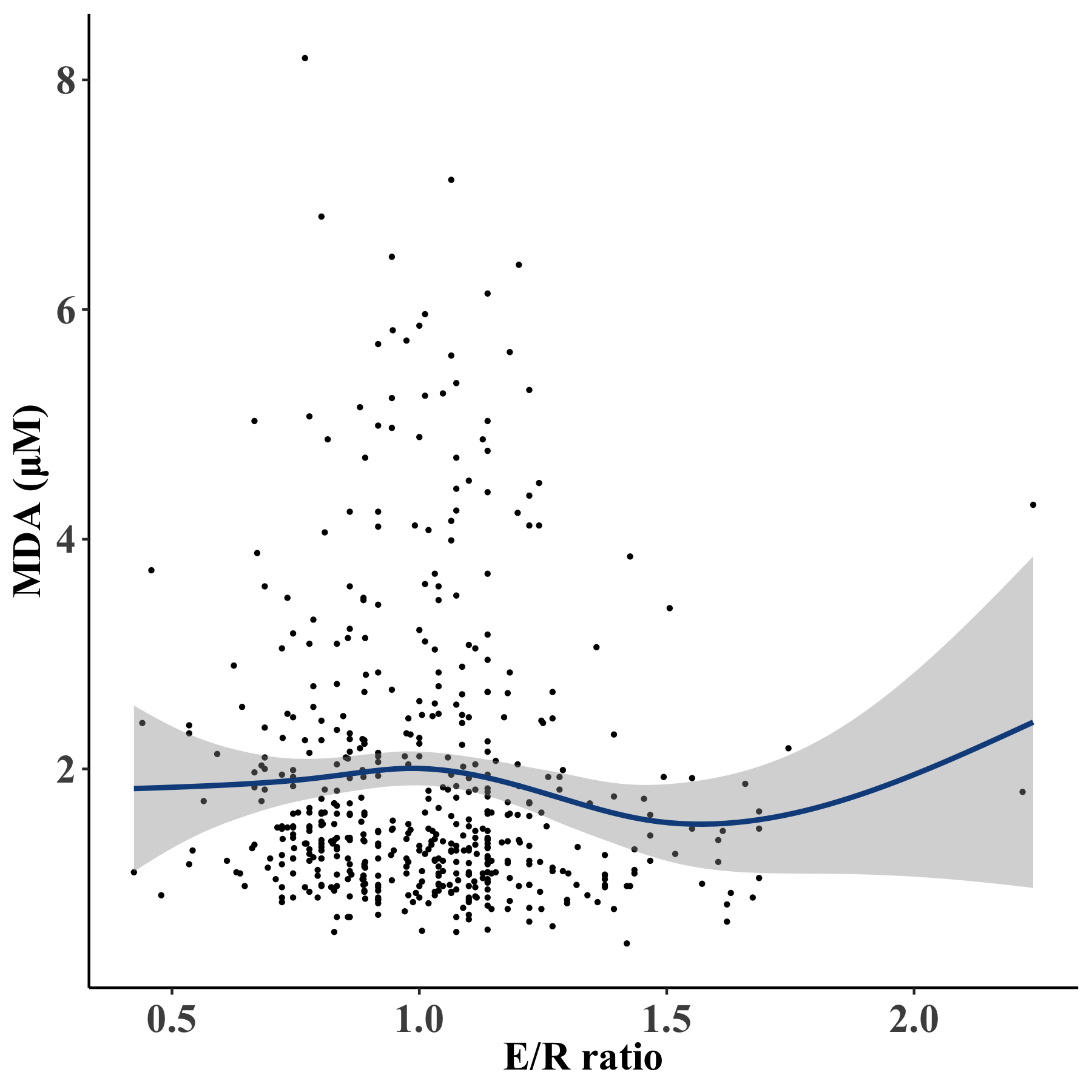

Supplement: Supplementary file 3 — Additional file 3: Fig. S3. Smooth curve fitting by the generalized additive model of MDA level and E/R ratio. [file 12888_2022_4226_MOESM3_ESM.tif]

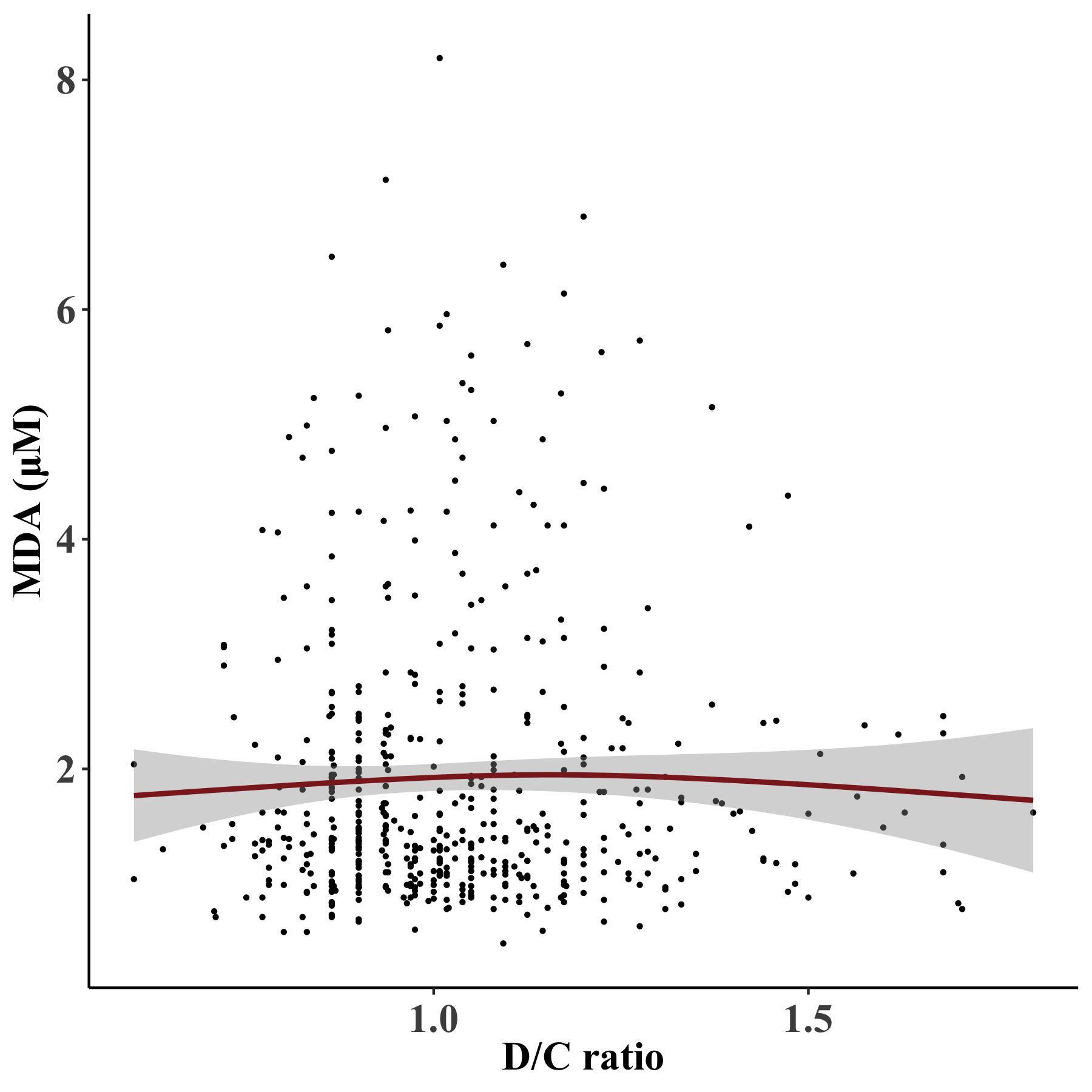

Supplement: Supplementary file 4 — Additional file 4: Fig. S4. Smooth curve fitting by the generalized additive model of MDA level and D/C ratio. [file 12888_2022_4226_MOESM4_ESM.tif]
